# Investigating the electronic properties of edge glycine/biopolymer/graphene quantum dots

**DOI:** 10.1038/s41598-024-71655-1

**Published:** 2024-09-20

**Authors:** Nayera M. El-Sayed, Hanan Elhaes, Asmaa Ibrahim, Medhat A. Ibrahim

**Affiliations:** 1 Physics Department, Faculty of Science, Mansoura University, Mansoura, 35516 Egypt; 2 Physics Department, Faculty of Women for Arts, Science and Education, Ain Shams University, Cairo, 11757 Egypt; 3 Spectroscopy Department, National Research Centre, 33 El-Bohouth St., Dokki, Giza, 12622 Egypt; 4 Molecular Modeling and Spectroscopy Laboratory, Centre of Excellence for Advanced Science, National Research Centre, 33 El-Bohouth St., Dokki, Giza, 12622 Egypt

**Keywords:** GQDs, Glycine, TDM, HOMO/LUMO and MESP, Materials science, Physics

## Abstract

This study systematically investigated four types of graphene quantum dots (GQDs) AHEX, ZTRI, ZHEX, and ATRI, and their interactions with glycine to form GQD-glycine complexes. Utilizing density functional theory (DFT) and the PM6 semiempirical method, the study analyzed electronic properties and structure-activity relationships. Global reactivity indices were calculated using Koopmans’ theorem, and quantitative structure-activity relationship (QSAR) parameters were assessed via SCIGRESS 0.3. The study further explored interactions using density of states (DOS) and quantum theory of atoms in molecules (QTAIM) analyses. Key findings revealed that glycine interaction significantly increased the total dipole moment (TDM) and decreased the HOMO/LUMO energy gap (ΔE) for the GQD-glycine complexes. Notably, ZTRI/glycine showed a TDM of 4.535 Debye and a reduced ΔE of 0.323 eV, indicating enhanced reactivity. Further interactions with cellulose, chitosan, and sodium alginate identified the ZTRI/glycine/sodium alginate composite as the most reactive, with a TDM of 8.020 Debye and the lowest ΔE of 0.200 eV. This composite also exhibited the highest electrophilicity index (56.421) and lowest chemical hardness (0.145 eV), underscoring its superior reactivity and stability. DOS analysis revealed that biomolecules contributed the most to molecular orbitals, with carbon atoms contributing the least. QTAIM analysis confirmed the greater stability of the ZTRI/glycine/sodium alginate complex compared to other studied composites. These results highlight the enhanced reactivity and stability of GQDs when interacting with glycine and sodium alginate.

## Introduction

Quantum dots, often referred to as QDs, represent a category of nanoscale semiconductor crystals^1^. Carbon-based substances like graphene have the potential to be utilized in the creation of Quantum Dots, leading to the formation of graphene quantum dots (GQDs)^2^. GQDs are defined as zero-dimensional carbon nanomaterials, have excellent fluorescence emission properties, and show excellent characteristics distinguished from conventional semiconductor quantum dots for their low cytotoxicity, good resistance to photobleaching, and excellent biocompatibility^3–5^. The development and understanding of GQDs have progressed significantly since their discovery. Various synthesis methods have been explored, including the one-step hydrothermal or solvent thermal processes, which are common despite their high temperature (over 180 °C) and long duration requirements that often result in the pyrolysis and polymerization of functional groups^6,7^. Alternative methods such as chemical vapor deposition, microwave-assisted synthesis, and electrochemical methods have also been investigated to optimize the properties and functionalities of GQDs^8^. Each synthesis method offers distinct advantages and challenges, contributing to the ongoing refinement of GQD production techniques. For instance, Liu et al. reported on the use of chemical vapor deposition for the synthesis of high-quality GQDs, which provided enhanced control over size and surface properties^9^.

Functionalization is a crucial approach for enhancing the surface characteristics and overall performance of GQDs^10,11^. By modifying the surface of GQDs, their applicability in various fields can be significantly improved. In recent years, amino acid-functionalized GQDs have shown a great attention in various applications^12,13^. This amino acid is uncomplicated, featuring amine and carboxyl groups attached to a carbon atom. Glycine was the first amino acid to be isolated from the hydrolysis of protein by Henri Braconnot in 1820^14,15^. It could be used to functionalize GQDs for environmental applications. In this sense, a facile post-modification method has been developed for the fabrication of glycine-functionalized graphene quantum dots (Gly-GQDs). The prepared Gly-GQDs showed efficient application for the determination of Hg^2+^ in fresh water^16^. Moreover, understanding how different conformations of glycine influence its interaction with GQDs is crucial for a comprehensive analysis of these complexes. Glycine can adopt various conformations due to its flexibility around the C-N bond^17^. These conformations can affect how glycine interacts with GQDs and consequently influence the properties of the GQD–glycine complexes^18^. In fact, based on the low cytotoxicity and chemical inertia of functionalized GQDs, they have several environmental applications. Dopamine-functionalized GQDs could be used as graphene quantum dots for successive detection of nanomolar ferric ions^19^. Gautam et al. demonstrated the use of GQDs functionalized with polyethylene glycol (PEG) for targeted drug delivery, showcasing their potential in therapeutic applications^20^.

GQDs could be further enhanced with Rhodamine to be used for the possible detection of Fe^3+^ in cancer stem cells^21^. This application paves the way toward applications of Rhodamine/GQDs in the field of bioimaging. Another way to apply GQDs in the field of bioimaging could be as AuNPs/glycine/GQDs composites with tunable bi-functionalities for cellular imaging^22^. Furthermore, Facile preparation was used for novel PEG-functionalized QDs with glycine-enhanced fluoroimmunoassays used to detect hazardous AFB1 in medicinal herbs^23^. Based on the phenomena of quantum confinement, the density of sp^2^ sites, and edge effects contribute to the unusual photoluminescence properties of GQDs, which makes them the best candidate for huge applications that could be summarized as biomarkers, drug delivery, bioimaging, biosensors, biolabeling, therapeutics, neuroscience, batteries, fuel cells, supercapacitors, electrochemical sensors, etc^24–28^. For example, Wang et al. successfully employed GQDs for in vivo bioimaging, highlighting their biocompatibility and effectiveness in tracking cellular processes^29^.

Computational methods, particularly quantum mechanical calculations and molecular modeling, are essential for investigating the electronic, physical, chemical, and biological properties of functionalized materials, including functionalized GQDs^30–32^. These techniques have been employed to study various nanoscale materials, providing valuable insights into their potential applications in biomedicine and other fields^33,34^. As mentioned earlier, the division of a graphene sheet into smaller clusters resulted in the identification of four distinct types of Graphene Quantum Dots (GQDs). These were classified as follows: hexagonal with armchair termination, referred to as AHEX; hexagonal with zigzag termination, termed ZHEX; triangular with zigzag termination, labeled ZTRI; and triangular GQD with armchair termination, known as ATRI^35^. It was also demonstrated that the shape and the imbalance in the number of atoms in GQDs lead to the existence of zero-energy states, and this shape is correlated with both the total and local magnetic moments^36^. A fluorescence sensor using glucosaminic acid/GQDs was prepared to be applied as it has high selectivity and sensitivity for lactose detection. Density functional theory (DFT) was used to elucidate the mechanism and confirm the experimental findings^37^.

The present work is conducted to study four types of glycine edge functionalized GQDs (AHEX; ZTRI; ZHEX and ATRI). Total dipole moment, HOMO/LUMO energy gap, and Molecular electrostatic potential MESP were calculated at B3LYP/6-31g(d,p). The reactivity was measured by global reactivity and QSAR descriptors. The most reactive type of GQDs will be further interacted through edge with cellulose, chitosan and sodium alginate. Some descriptors will be presented for the studied structures.

## Computational methodology and justification

The model molecules were optimized using the Gaussian 09 (G09) software package^38^, implemented at the molecular modeling and spectroscopy laboratory, centre of excellence for advanced science, national research centre (NRC), Egypt. The optimization was performed using density functional theory (DFT) with the B3LYP functional and the 6-31G(d,p) basis set^39–41^. This level of theory was selected for its balance between accuracy and computational efficiency, and it is well-established for predicting molecular geometries and electronic properties. Subsequently, molecular properties such as the total dipole moment, HOMO/LUMO energy gap, and molecular electrostatic potential (MESP) were computed using the same DFT/6-31G(d,p) method. These properties are crucial for understanding the reactivity and interaction potentials of the model molecules. Although the DFT/B3LYP method is generally reliable for these calculations, it may have limitations in accurately predicting weak interactions, which are inherently more challenging to model. This method was chosen due to its wide application in the literature and its efficacy in providing insights into molecular behavior^42^.

To compute global reactivity indices such as electron affinity (A), ionization energies (I), electrophilicity index (ω), nucleophilicity index (ε), chemical potential (μ), electronegativity (χ), hardness (η), and softness (S), we applied Koopmans’ theorem in conjunction with frontier molecular orbitals using the Spartan software, employing the 6-31+G** basis set^43^. The descriptors were selected based on their established use in predicting reactivity trends in molecular systems. For quantitative structure-activity relationship (QSAR) analysis, we examined the effects of chemical structure on interactions with cellulose, chitosan, and sodium alginate. Parameters such as accessible surface area (Acc. Area), polar surface area (PSA), accessible polar surface area (Acc. P-area), and electrostatic potentials (Min ElPot and Max ElPot) were calculated using SCIGRESS 0.3 and the PM6 semiempirical method^44^. These QSAR calculations were instrumental in exploring the interaction dynamics of the ZTRI/glycine composite with the biomaterials. The Quantum Theory of Atoms in Molecules (QTAIM) analysis was performed on the examined structures to further investigate the interactions between graphene quantum dots and the biomaterials. This analysis was conducted using the "output=wfn" command in the Gaussian software, with visualization of the results facilitated by Avogadro software^45^.

The chosen computational methods, including DFT/B3LYP for electronic properties and semiempirical methods for QSAR analysis, were selected for their reliability and common use in similar studies. However, these methods may not fully capture weak interactions, and the study's focus on four specific types of graphene quantum dots (GQDs) and their interactions with glycine and selected biomaterials may not cover the full range of GQD properties. Future research should explore a broader range of GQD types and their interactions with different amino acids. To improve accuracy, additional computational methods, such as higher-level ab initio calculations or dispersion-corrected DFT, should be considered, alongside comparative studies at different theoretical levels. Further investigations will also examine the impact of glycine conformations on GQD properties.

## Results and discussions

### Building model molecules

**Fig. 1:**
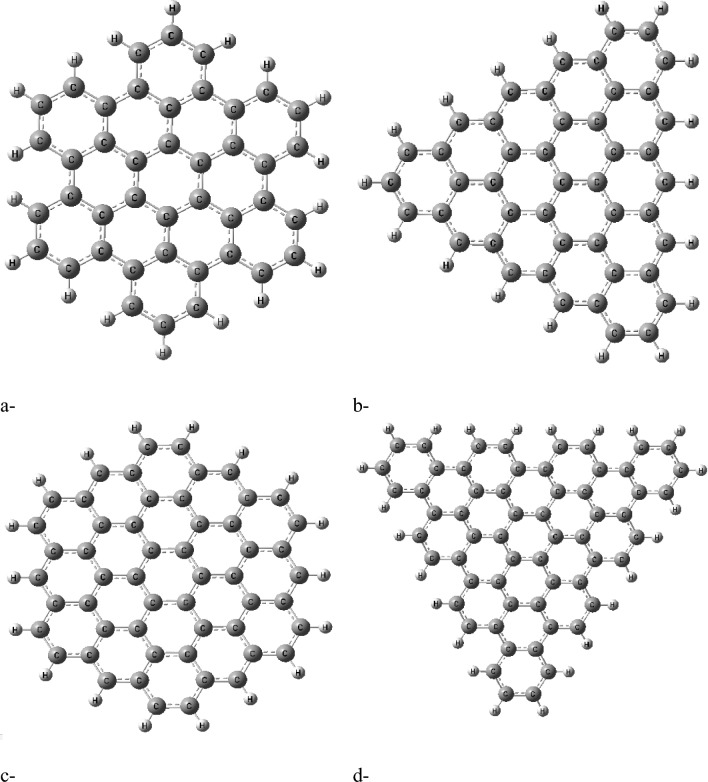
Studied graphene quantum dots GQD whereas (**a**) AHEX; (**b**) ZTRI; (**c**) ZHEX and (**d**) ATRI.

In this study, four types of graphene quantum dots (GQDs) were investigated: AHEX, ZTRI, ZHEX, and ATRI, as depicted in Figure 1a–d, respectively. Glycine was studied for its interaction with each GQD type, hypothesized to form GQD–amino acid complexes through edge interactions. This approach is consistent with the findings of Wang et al., who demonstrated that the edge sites of GQDs play a crucial role in interaction with small molecules, highlighting their potential for forming stable complexes^46^. For each optimized structure, several key physical parameters were calculated: the total dipole moment (TDM), the HOMO/LUMO energy gap, and the molecular electrostatic potential (MESP). Based on the physical parameters and their comparison with previous research, the most reactive GQD composite was selected for further interaction studies with biomaterials such as cellulose, chitosan, and sodium alginate.

### Calculated physical parameters

The physical parameters calculated include the total dipole moment (TDM), HOMO/LUMO energy gap, and molecular electrostatic potential (MESP), which are critical for assessing the reactivity and stability of the GQD–glycine complexes. The TDM measures the separation of positive and negative charges within a molecule, reflecting its polarity and potential for interaction with other molecules^47^. According to the findings presented in Table 1, the calculated TDMs for the pure GQDs AHEX, ZTRI, ZHEX, and ATRI were 0.000 Debye, indicating no significant dipole moment for these GQDs in their pure form.

**Table 1:** B3LYP/6-31g(d,p) calculated total dipole moment (TDM) as Debye; HOMO/LUMO energy gap (∆E) as eV for the studied GQDs with their glycine composite.

Structure	TDM (Debye)	∆E (eV)
AHEX	0.000	3.589
AHEX/glycine	3.593	3.396
ZTRI	0.000	0.322
ZTRI/glycine	4.535	0.323
ZHEX	0.000	2.821
ZHEX/glycine	4.189	2.714
ATRI	0.000	3.193
ATRI/glycine	4.301	3.071

The HOMO/LUMO gap is a key indicator of a material’s electronic properties and reactivity. A smaller HOMO/LUMO gap implies a higher tendency for electron transfer processes, which is essential for chemical reactivity^48^. The HOMO/LUMO gaps for GQDs were as follows: 3.589 eV (AHEX), 0.322 eV (ZTRI), 2.821 eV (ZHEX), and 3.193 eV (ATRI). Previous research supports the notion that an increased TDM and a reduced HOMO/LUMO energy gap enhance a compound's reactivity. For example, Lee et al. (2021) found that GQDs with a higher TDM exhibited greater interaction potentials with surrounding media. Similarly, Kondratenko et al. demonstrated that a lower HOMO/LUMO energy gap correlates with increased reactivity in carbon-based nanomaterials^49^. Upon interaction with glycine, the total dipole moments of the GQD–glycine composites increased, and the HOMO/LUMO energy gaps decreased. Specifically, the ZTRI/glycine composite showed a TDM of 4.535 Debye and a HOMO/LUMO energy gap of 0.323 eV, as reported in Table 1. This significant increase in TDM and the substantial decrease in the HOMO/LUMO energy gap suggest that ZTRI/glycine is the most reactive composite among the four studied. The increased reactivity of ZTRI/glycine can be attributed to its enhanced ability to interact with surrounding media, as indicated by its high TDM and low energy gap. Additionally, The TDM varied significantly with different glycine conformations. For instance, Mahmud et al. found that the zwitterionic form of glycine exhibited higher dipole moments when interacting with GQDs compared to its neutral form^50^. This is attributed to the presence of both positive and negative charges in the zwitterionic state, which enhances the overall dipole of the complex.

**Fig. 2:**
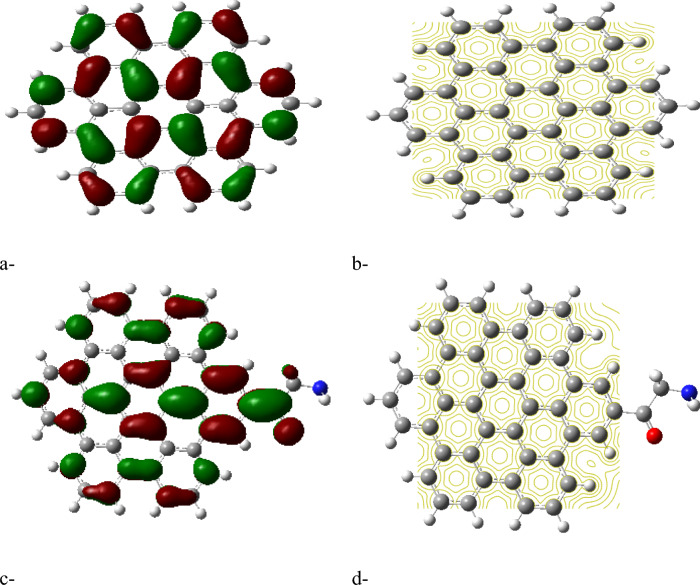
B3LYP/6-31g (d, p) calculated (**a**) HOMO/LUMO energy gap for AHEX; (**b**) MESP for AHEX; (**c**) HOMO/LUMO energy gap for AHEX/glycine and (**d**) MESP for AHEX/glycine.

**Fig. 3:**
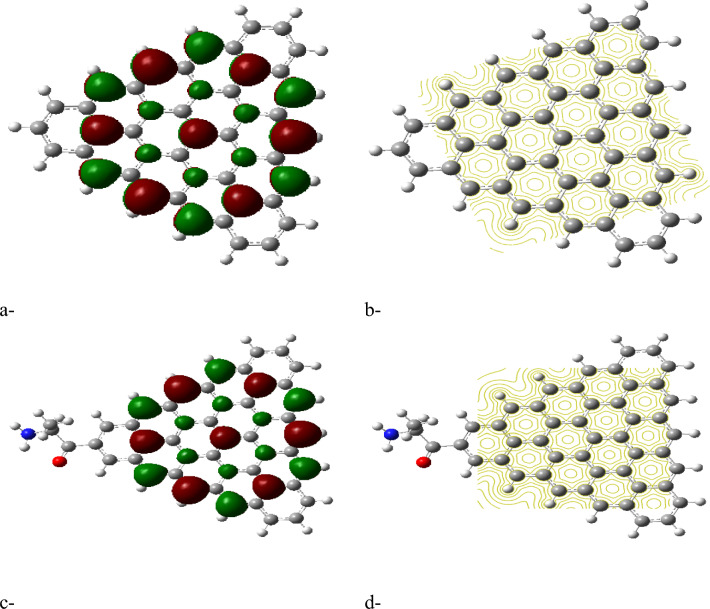
B3LYP/6-31g (d, p) calculated (**a**) HOMO/LUMO energy gap for AHEX; (**b**) MESP for ZTRI; (**c**) HOMO/LUMO energy gap for ZTRI/glycine and (**d**) MESP for ZTRI/glycine.

**Fig. 4:**
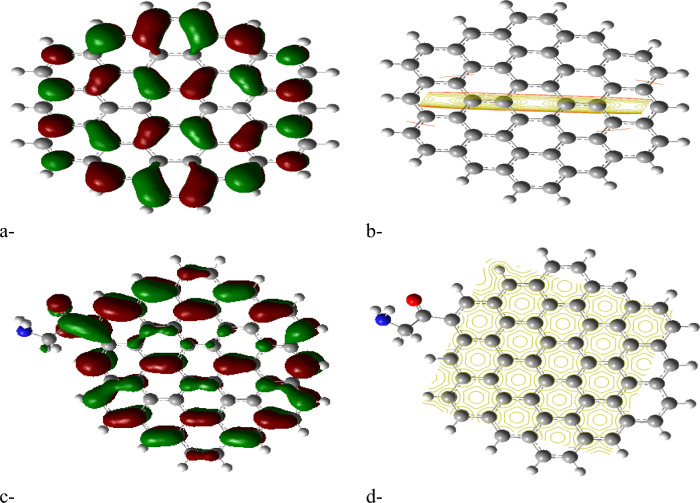
B3LYP/6-31g (d, p) calculated (**a**) HOMO/LUMO energy gap for AHEX; (**b**) MESP for ZHEX; (**c**) HOMO/LUMO energy gap for ZHEX/glycine and (**d**) MESP for ZHEX/glycine.

**Fig. 5:**
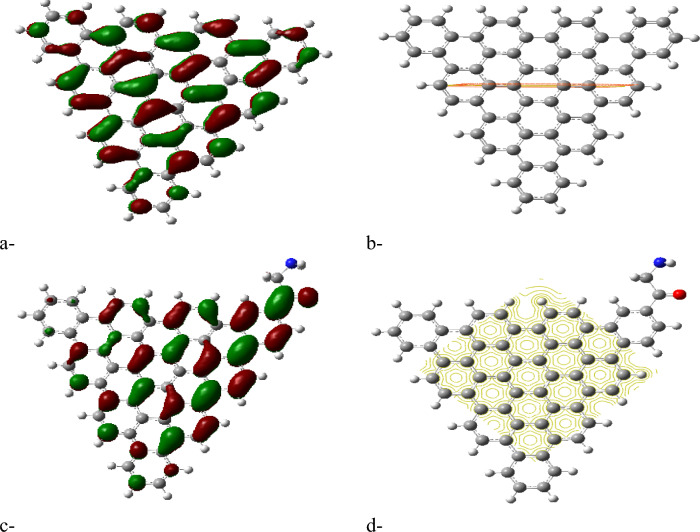
B3LYP/6-31g(d,p) calculated (**a**) HOMO/LUMO energy gap for ATRI; (**b**) MESP for ATRI; (**c**) HOMO/LUMO energy gap for ATRI/glycine and (**d**) MESP for ATRI/glycine.

The HOMO/LUMO energy gap calculations, shown in Figures 2a, 3a, 4a and 5a, reveal that the ZTRI type exhibits the highest dipole moment, suggesting a greater potential for reactivity compared to the other GQD types. This observation is in agreement with the results reported by Sebastian et al., who noted that a higher dipole moment often correlates with increased reactivity in GQD-based systems^51^. The analysis of the HOMO/LUMO energy gap for the proposed complexes reveals that ZTRI/glycine has the lowest energy gap (as depicted in Figures 2c, 3c, 4c, and 5c), suggesting it is most prone to facilitate electron transfer processes. This observation is consistent with Henna et al. who found that a reduced energy gap in GQDs can improve their electronic interactions with biomolecules^52^. Additionally, various studies reveal that different glycine conformations impact the HOMO/LUMO energy gap of the GQD–glycine complexes. Larijani et al. also observed that the zwitterionic form of glycine typically results in a lower HOMO/LUMO gap, indicating heightened reactivity^53^.

**Fig. 6:**
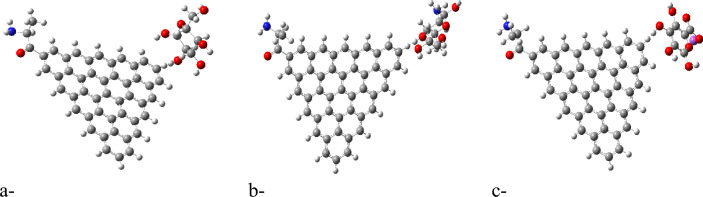
Model molecules for ZTRI/glycine with (**a**) cellulose, (**b**) chitosan and (**c**) sodium alginate.

Molecular electrostatic potential (MESP) provides insight into the electrostatic environment around a molecule, indicating regions of positive or negative potential^54^. The MESP maps for each GQD and its glycine composite are illustrated in Figures 2b,d up to 5b,d. The MESP is represented in a color scheme where yellow indicates neutrality, neither positive nor negative. The GQDs in their neutral forms are shown in yellow, while the glycine interaction results in a more uniform potential distribution across the GQD surfaces. The MESP data highlight that the ZTRI type exhibits a uniform MESP both before and after interaction with glycine. This uniformity suggests that ZTRI maintains consistent electrostatic interactions, making it a suitable candidate for further studies. This observation is supported by the findings of Ibrahim et al., who reported that uniform MESP can enhance interaction with other biomolecules^55^. The ZTRI/glycine composite was selected for further interaction studies with biomaterials such as cellulose, chitosan, and sodium alginate. Figure 6a–c illustrates the interactions of the ZTRI/glycine complex with these biomaterials, respectively. The glycine interacts at one edge of the ZTRI structure, while the biomaterials interact at the opposite edge.

**Fig. 7:**
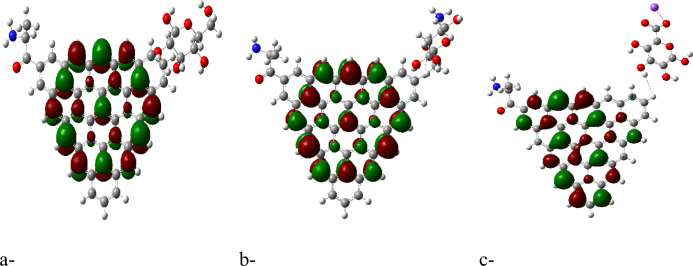
HOMO/LUMO energy gap for ZTRI/glycine with (**a**) cellulose, (**b**) chitosan and (**c**) sodium alginate.

**Fig. 8:**
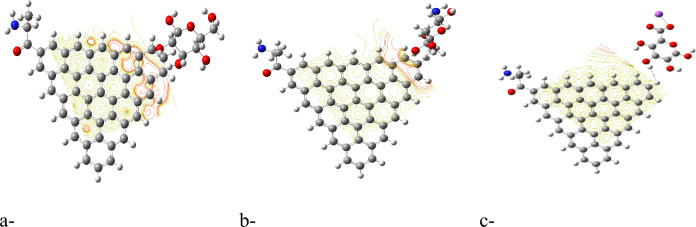
Molecular electrostatic potential MESP for ZTRI/glycine with (**a**) cellulose, (**b**) chitosan and (**c**) sodium alginate.

Figure 7a–c presents the mapped HOMO/LUMO energy gaps for ZTRI/glycine interacting with cellulose, chitosan, and sodium alginate, respectively. The HOMO/LUMO mapping in Figure 7 demonstrates that ZTRI/glycine exhibits uniform distribution of HOMO and LUMO orbitals across the ZTRI surface for both ZTRI/glycine/cellulose and ZTRI/glycine/chitosan interactions. This uniform distribution suggests stable interactions with these biomaterials. Figure 8a–c shows the MESP for ZTRI/glycine interacting with the same biomaterials. The MESP maps reveal a uniform potential in the center of the ZTRI structure (yellow). Edges close to the biopolymer show a red color, indicating its ability for further interaction throughout the edge, which is an indication of the ability of the biopolymer to enhance the ZTIR structure throughout the ring near the edge. The decreased TDM and ΔE for sodium alginate suggest that while the interaction is strong, it is less uniform compared to cellulose and chitosan.

Table 2 presents the B3LYP/6-31G(d,p) calculated values for the total dipole moment (TDM) and HOMO/LUMO energy gap (ΔE) for the ZTRI/glycine composite interacting with cellulose, chitosan, and sodium alginate. For the ZTRI/glycine composite interacting with cellulose, the TDM was found to be 3.523 Debye, and the HOMO/LUMO energy gap was 5.663 eV. These values suggest a moderate reactivity. In contrast, for the interaction with chitosan, the TDM decreased to 1.151 Debye, and ΔE was 4.753 eV, indicating a reduction in reactivity. When interacting with sodium alginate, the TDM was 3.627 Debye and ΔE was 1.647 eV, showing a variable reactivity depending on the biomaterial. Notably, the TDM for the ZTRI/glycine composite itself was 4.535 Debye with a reduced ΔE of 0.323 eV, suggesting high reactivity. When interacting with cellulose, the TDM decreased to 2.763 Debye, and ΔE increased to 1.264 eV. In the case of ZTRI/glycine/chitosan, the TDM increased to 4.867 Debye, and ΔE decreased to 0.330 eV, indicating enhanced reactivity. For ZTRI/glycine/sodium alginate, the TDM significantly decreased to 8.020 Debye, while ΔE was the lowest at 0.200 eV, suggesting the highest reactivity among the studied composites. The significant variation in TDM and ΔE values among different biomaterials indicates that the interaction strength and reactivity of ZTRI/glycine are highly dependent on the biomaterial used. Based on the values of both TDM and ΔE, the ZTRI/glycine composite interacting with sodium alginate exhibits the highest reactivity among the studied composites. This conclusion is supported by the consistent MESP and HOMO/LUMO mapping results, which indicate strong and stable interactions, particularly with sodium alginate.

**Table 2:** B3LYP/6-31g(d,p) calculated total dipole moment (TDM) as Debye; HOMO/LUMO energy gap (∆E) as eV for the studied ZTRI/glycine composite interacted with cellulose, chitosan and sodium alginate.

Structure	TDM (Debye)	∆E (eV)
Cellulose	3.523	5.663
Chitosan	1.151	4.753
Sodium alginate	3.627	1.647
ZTRI/glycine	4.535	0.323
ZTRI/glycine/cellulose	2.763	1.264
ZTRI/glycine/chitosan	4.867	0.330
ZTRI/glycine/sodium alginate	8.020	0.200

### Calculated reactivity descriptors

Global reactivity descriptors, indicated how easily the studied molecules loss or gain electrons (ionization potentials and electron affinities) using different methods and a specific set of parameters (at 6–31+G** basis set). The results are presented in Table 3. Ionization energy is a measure of how easily a molecule loses an electron, which is linked to its chemical reactivity. Generally, high ionization energy means a molecule is stable and unreactive, while low ionization energy indicates high reactivity^56^. In this study, the addition of various materials (cellulose, chitosan, and sodium alginate) to a composite material (ZTRI/glycine) caused changes in its ionization energy (IP). Interestingly, adding sodium alginate increased the calculated IP as shown in Table 3, which suggests it might be more reactive than the other components. This finding is consistent with the work of Domingo et al., who found that increased IP can indicate enhanced reactivity due to the stabilization effects of the added material^57^.

**Table 3:** Calculated HOMO, LUMO energy, global reactivity descriptors for the studied ZTRI/glycine composite interacted with cellulose, chitosan and sodium alginate using the B3LYP/6-31g(d,p) model.

Structure	LUMO	HOMO	Ionization potential (I)	Electronic affinity (A)	Electronic chemical potential (μ)	Chemical hardness (η)	Absolute softness (S)	Electrophilicity index (ω)
Cellulose	− 1.46	− 7.12	7.12	− 1.46	− 2.83	4.29	0.233	0.933
Chitosan	− 1.82	− 6.57	6.57	− 1.82	− 2.38	4.20	0.238	0.672
Sodium alginate	− 1.28	− 2.93	2.93	1.28	− 2.11	0.83	1.21	2.69
ZTRI/glycine	− 3.65	− 3.98	3.98	3.65	− 3.81	0.17	2.93	44.10
ZTRI/glycine/cellulose	− 3.19	− 4.40	4.40	3.19	− 3.80	0.60	0.83	11.90
ZTRI/glycine/chitosan	− 3.63	− 3.96	3.96	3.63	− 3.80	0.17	2.93	43.64
ZTRI/glycine/sodium alginate	− 3.90	− 4.19	4.19	3.90	− 4.05	0.15	3.33	56.42

Electron affinity denotes the ability of a ligand to selectively receive a single electron from a donor^58^. A positive electron affinity signifies the release of energy upon electron addition, indicating the favorability of the atom or molecule to accept an electron. Conversely, a negative electron affinity implies that energy input is required for electron incorporation into the system. The electron affinities of the ZTRI/glycine composite were calculated in interaction with cellulose, chitosan, and sodium alginate (refer to Table 3). The findings reveal that the ZTRI/glycine composite in interaction with sodium alginate exhibits the highest electron affinity value, while cellulose displays a negative electron affinity value. These observations align with the findings of Kim et al., who demonstrated that higher electron affinity often correlates with better electron acceptance and interaction potential^59^. The high electron affinity of the ZTRI/glycine/sodium alginate composite implies that it may be more reactive and capable of engaging in electron transfer processes compared to composites with cellulose or chitosan.

The electronic chemical potential (μ) was also determined, which represents the energy needed to add or remove an electron from a system while maintaining constant volume and temperature. Additionally, a molecule characterized by a substantial energy gap is termed "hard," while one with a small energy gap is termed "soft." It's important to note that hard molecules are less polarizable compared to soft ones, as they demand significant energy for excitation^60^. Utilizing Koopmans' theorem for closed-shell compounds, the electronic chemical potential (μ), chemical hardness (η), and absolute softness (S) can be formally defined.

$$\mu =\frac{-(I+A)}{2},$$

$$\eta =\frac{1-A}{2},$$

$$S=\frac{1}{2\eta },$$

where I and A are the ionization potential and electron affinity of the compounds respectively.

Absolute hardness and softness serve as crucial indicators for evaluating the stability and reactivity of molecules. Chemical hardness, in particular, denotes the inherent resistance to the deformation or polarization of the electron cloud within atoms, ions, or molecules when subjected to minor perturbations in chemical reactions. In the present investigation, the ZTRI/glycine composite exhibited a hardness value of 0.17 eV and a chemical potential of -3.81 eV (refer to Table 3). Notably, the hardness value and chemical potential of this composite decreased following its interaction with the suggested molecules, indicating an enhancement in chemical reactivity, particularly in the case of sodium alginate. These findings align with previous studies, such as those by Demircioğlu et al., which demonstrated that a decrease in hardness and chemical potential is associated with enhanced reactivity in molecular systems^61^.

Parr et al.^62^ introduced a novel descriptor to quantify the overall electrophilic potency of a compound, termed the electrophilicity index (ω). This index establishes a quantitative classification of a compound's global electrophilic nature. The electrophilicity index (ω) proposed by Parr et al. was designed to gauge the energy reduction resulting from the maximal electron flow between a donor and an acceptor. The definition of the electrophilicity index (ω) by Parr et al. is as follows

$$\omega =\frac{{\mu }^{2}}{2\eta }.$$

The recent application of this novel reactivity metric has proven instrumental in comprehending the toxicity of diverse pollutants concerning their reactivity and site selectivity. The computed electrophilicity index value characterizes the biological activity of the ZTRI/glycine composite when interacting with cellulose, chitosan, and sodium alginate. In this study, the ZTRI/glycine composite showed varying electrophilicity indices based on its interactions with different biomaterials. Specifically, the ZTRI/glycine/sodium alginate composite exhibited the highest electrophilicity index, reflecting its strong electrophilic character. This result supports the work of Parr et al., who found that a higher electrophilicity index correlates with greater electrophilic potency and reactivity.

### Calculated quantitative structure-activity relationship (QSAR) descriptors

QSAR was calculated to examine how the chemical structure of a compound made from ZTRI and glycine affects its interactions with cellulose, chitosan, and sodium alginate. To do this, specific features of the compound, such as accessible surface area (Acc. Area), Polar surface area (PSA), the accessible polar surface area (Acc. P-area), the minimum values of the electrostatic potential (Min ElPot), the maximum values of the electrostatic potential (Max ElPot), the minimum value of the local ionization potential (Min LocionPot), were calculated. These features are like measurable characteristics that help describe the ZTRI/glycine composite's interaction abilities. The QSAR outcomes for the recommended compounds are documented in Table 4**.**

**Table 4:** QSAR descriptors calculated at B3LYP/6-31G(d, p) for the studied ZTRI/glycine composite interacted with cellulose, chitosan and sodium alginate.

Structure	Volume (Å^3^)	Acc. Area (Å^2^)	(PSA) (Å^2^)	Acc. P-area (Å^2^)	Min ElPot (KJ/mol)	Max ElPot (KJ/mol)	Min LocionPot (KJ/mol)
Cellulose	158.13	112.67	65.28	50.00	− 182.65	263.00	39.99
Chitosan	161.42	115.51	78.34	56.23	− 187.34	220.62	34.52
Sodium alginate	154.97	140.44	103.91	80.73	− 317.15	551.99	36.60
ZTRI/glycine	625.90	430.95	56.68	42.31	− 215.76	118.42	29.51
ZTRI/glycine/cellulose	781.87	508.09	131.82	95.24	− 205.99	272.95	19.94
ZTRI/glycine/chitosan	786.35	514.62	121.24	86.03	− 218.64	223.90	29.49
ZTRI/glycine/sodium alginate	781.17	544.97	131.97	97.78	− 303.67	565.27	28.72

A significant rise in the volume of the ZTRI/glycine composite was noticed when cellulose, chitosan, and sodium alginate were introduced. This alteration encourages us to explore potential interactions or impacts arising from the incorporation of these additional compounds into the ZTRI/glycine composite. The term "Acc. Area" pertains to the portion of a molecule's surface accessible to solvent molecules, considering its three-dimensional structure and the space available for solvent occupancy. Acc. Area calculations are commonly employed to forecast properties such as solubility and interactions with other molecules^63^. The enrichment of QSAR dataset with supplementary molecules for the ZTRI/glycine composite has revealed a substantial increase in Acc. Area. This interesting observation prompts a more in-depth investigation into the molecular dynamics and interactions underlying this expansion. The heightened accessible surface area indicates a broader molecular exposure of the ZTRI/glycine composite to its surroundings upon the addition of cellulose, chitosan, and sodium alginate. This could potentially influence intermolecular interactions and reactivity.

The polar surface area (PSA) specifically denotes the surface area of a molecule occupied by polar atoms^64^. On the other hand, the Acc. P area combines the concepts of accessible surface area and polar surface area, representing the portion of a molecule's surface area that is both accessible to solvent molecules and occupied by polar atoms^65^. Our QSAR results have revealed a significant increase in both PSA and Acc. P area for the ZTRI/glycine composite, particularly evident when incorporating the suggested molecules, especially in the case of ZTRI/glycine/sodium alginate. This noteworthy discovery underscores the heightened polar character introduced by the additional compounds, suggesting potential modifications in intermolecular interactions. The observed expansion indicates an increased exposure of polar functionalities within the molecular structures of the ZTRI/glycine composite, hinting at potential consequences for intermolecular interactions and the modulation of biological activities.

Electrostatic potential, defined as the electric potential energy per unit charge at a specific point surrounding a molecule, serves as a crucial measure in molecular analysis^66^. Min ElPot is typically situated in regions abundant in electron density, where a positive test charge experiences attraction. Conversely, Max ElPot is found in regions with electron deficiency, causing repulsion for a positive test charge. The expansion of our QSAR dataset, incorporating additional molecules, has brought about noticeable alterations in both Min ElPot and Max ElPot. This intriguing development indicates shifts in electronic distributions within the molecular structures, hinting at potential changes in the reactivity and intermolecular interactions of the ZTRI/glycine composite following the addition of cellulose, chitosan, and sodium alginate. The local ionization potential, on the other hand, focuses on the energy required to remove an electron from a specific location within a molecule^67^. Min LocionPot denotes the minimum ionization potential at a specific location within a molecule. A minimum local ionization potential indicates that less energy is required to remove an electron from that specific region compared to neighboring areas. This information is crucial for comprehending the reactivity and chemical behavior of distinct parts of a molecule. The decrease in the minimum local ionization potential of the ZTRI/glycine composite subsequent to the addition of cellulose, chitosan, and sodium alginate prompts a thorough investigation into how the introduced molecules contribute to the adjustment of electron affinities within our dataset and the potential implications for the predictive accuracy of our QSAR model.

### DOS and QTAIM analyses

**Fig. 9:**
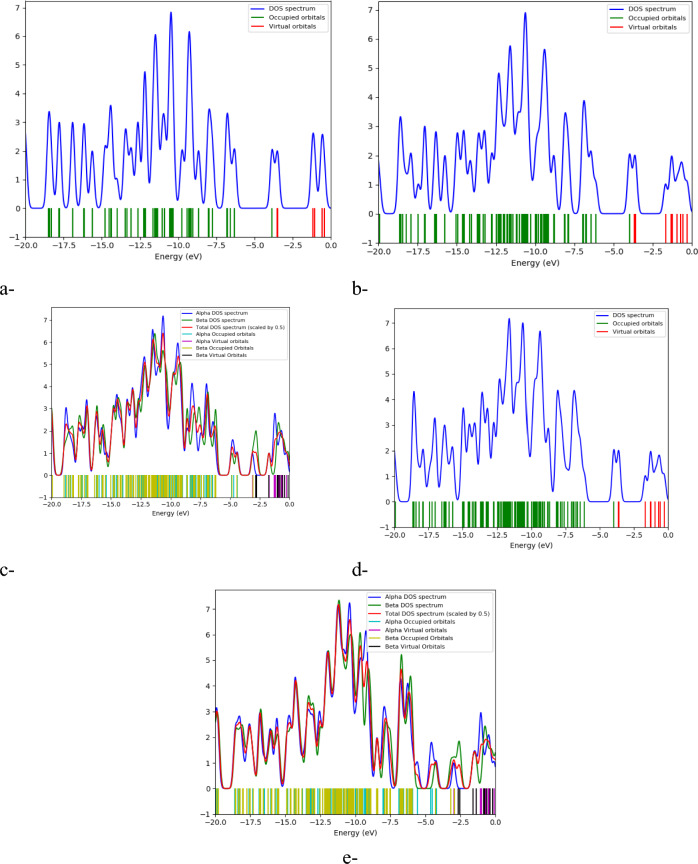
Density of states (DOS) analysis for (**a**) ZTRI, (**b**) ZTRI/glycine, (**c**) ZTRI/glycine/cellulose, (**d**) ZTRI/glycine/chitosan, and (**e**) ZTRI/glycine/sodium alginate.

The Density of States (DOS) and Quantum Theory of Atoms in Molecules (QTAIM) analyses provide significant insights into the electronic properties and molecular interactions within the studied systems. Figure 9 illustrates the DOS for the investigated structures: Figure 9a corresponds to ZTRI, Figure 9b to ZTRI/glycine, Figure 9c to ZTRI/glycine/cellulose, Figure 9d to ZTRI/glycine/chitosan, and Figure 9e to ZTRI/glycine/sodium alginate.

The DOS analysis shows the number of allowable states or modes per unit of energy for these structures. Notably, the HOMO levels were pushed closer to the Fermi level, indicating stronger molecular interactions between ZTRI and the biomolecules. This shift suggests an increased probability of electron transfer, as observed in similar studies, where graphene-based systems showed significant electronic reconfiguration upon interaction with biomolecules. For instance, in graphene/sodium alginate systems, the highest contribution to the molecular orbitals is attributed to sodium alginate, while carbon atoms exhibit the lowest contribution^68^. This pattern is consistent with our findings, where sodium alginate, compared to cellulose and chitosan, induces a more pronounced electron redistribution upon interacting with graphene. This redistribution is crucial for understanding the molecular reactivity and stability of the composites.

**Fig. 10:**
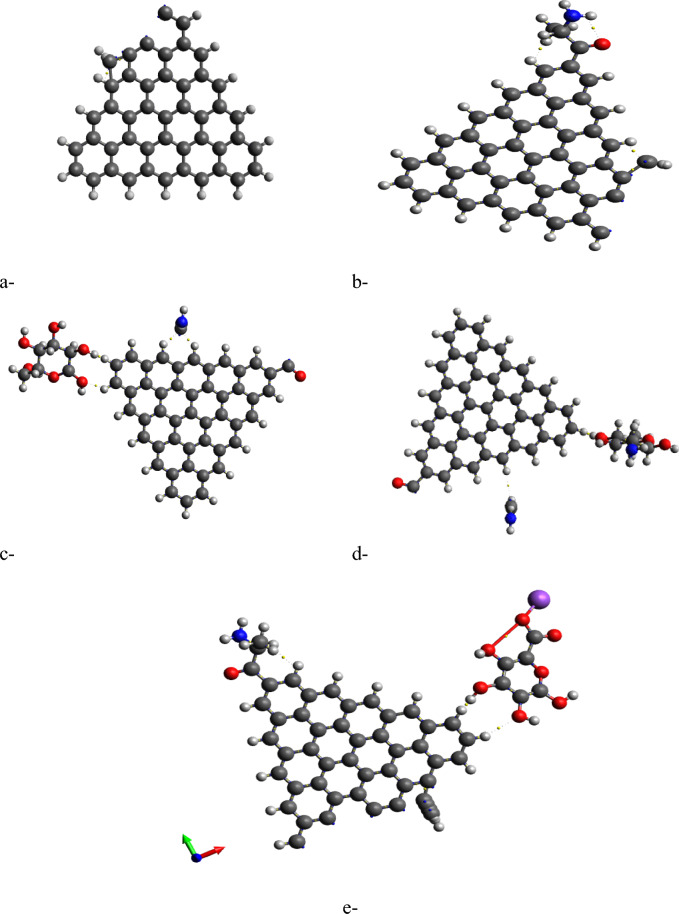
QTAIM analysis for (**a**) ZTRI, (**b**) ZTRI/glycine, (**c**) ZTRI/glycine/cellulose, (**d**) ZTRI/glycine/chitosan, and (**e**) ZTRI/glycine/sodium alginate.

QTAIM, a well-established methodology, provides detailed insights into the electronic density distribution, identifying bond paths and critical points that correspond to both bonds and bond orders^69^. In addition, it is a valuable tool for understanding adsorption processes and predicting the behavior of different molecules on various surfaces^70^. Within QTAIM analysis, the electron density at bond critical points (BCPs) between interacting atoms is essential for determining the strength of bonding interactions^71^. Higher electron density values at these critical points generally indicate stronger electronic charge densities, which correspond to more robust and covalent interactions. Additionally, if ∇2ρ(r) is less than 0 and H(r) is less than 0, it suggests a covalent (shared) interaction. In contrast, when ∇2ρ(r) is greater than 0 and H(r) is greater than 0, it points to non-covalent (closed-shell) interactions, such as weak hydrogen bonds, van der Waals forces, and electrostatic interactions. As illustrated in Figure 10, QTAIM analysis reveals the nature of non-covalent interactions within the studied structures. The analysis shows that ZTRI/glycine/sodium alginate (Figure 10e) is more stable than ZTRI/glycine/cellulose (Figure 10c) and ZTRI/glycine/chitosan (Figure 10d) structures. This stability is linked to stronger hydrogen bonding and van der Waals interactions, which are supported by higher values of electron density (ρ) and Laplacian of electron density (∇^2^ρ) at bond critical points, indicating stronger intermolecular forces.

The increased stability of the ZTRI/glycine/sodium alginate system aligns with previous findings where edge-functionalized graphene showed enhanced reactivity when blended with sodium alginate, compared to other polysaccharides^72^. This is due to sodium alginate’s ability to stabilize the composite through multiple non-covalent interactions, such as electrostatic interactions and hydrogen bonding, which are more prominent in the sodium alginate structure. Additionally, our results show that non-covalent interactions between ZTRI and glycine (Figure 10b) are significant, indicating a substantial role of glycine in modulating the overall electronic environment of the composite. In summary, the DOS and QTAIM analyses highlight the impact of molecular interactions on the electronic structure and stability of the composites. The findings underline the importance of selecting suitable biomolecules, such as sodium alginate, to enhance the properties of graphene-based materials for potential applications.

## Conclusion

The current computational investigation provides a comprehensive investigation of the interactions between four types of graphene quantum dots (GQDs)—AHEX, ZTRI, ZHEX, and ATRI—and glycine, with further interactions involving biomaterials such as cellulose, chitosan, and sodium alginate. Key physical parameters, including total dipole moment (TDM), HOMO/LUMO energy gap, and molecular electrostatic potential (MESP), were calculated for each GQD and its glycine complexes. The results indicate that the ZTRI/glycine composite exhibits the highest reactivity, as evidenced by its significant TDM increase to 4.535 Debye and a reduction in the HOMO/LUMO energy gap to 0.323 eV. This high reactivity is further supported by consistent and uniform MESP mapping, suggesting strong interaction potential with biomaterials. The ZTRI/glycine composite's interactions with cellulose, chitosan, and sodium alginate were examined, with sodium alginate showing the highest reactivity, indicated by the lowest HOMO/LUMO energy gap of 0.200 eV and a TDM of 8.020 Debye. Further analysis of global reactivity descriptors revealed that the ZTRI/glycine composite's interaction with sodium alginate leads to enhanced reactivity. QSAR descriptors also demonstrated an increase in accessible surface area (Acc. Area), polar surface area (PSA), and electrostatic potential, particularly with sodium alginate, highlighting the potential for strong intermolecular interactions.

The density of states (DOS) analysis revealed that the contributions to molecular orbitals varied among the studied structures, with the most significant contributions from the biomolecules, aligning with previous observations in graphene/sodium alginate systems. Quantum theory of atoms in molecules (QTAIM) analysis provided further insights, showing that the ZTRI/glycine/sodium alginate complex was more stable than the corresponding complexes with cellulose or chitosan. This suggests that blending edge-functionalized graphene with sodium alginate enhances reactivity more effectively than with other polysaccharides. Future research will extend these findings by exploring a broader spectrum of GQD types and glycine conformations, employing additional computational methods for verification, and conducting experimental validation through spectroscopy or microscopy. This will help to further elucidate the impact of various modifications on GQD reactivity and interactions with biomolecules, ultimately tailoring specific properties for diverse applications. In conclusion, this study underscores the significant influence of glycine conformations on GQD properties, laying the groundwork for future explorations and applications of functionalized GQDs in various fields.

## Data Availability

The data that support the findings of this study are available from the corresponding author upon reasonable request.
